# Improving signal-to-noise ratio for the forensic analysis of glass using micro X-ray fluorescence spectrometry

**DOI:** 10.1002/xrs.3179

**Published:** 2020

**Authors:** Ruthmara Corzo, Eric Steel

**Affiliations:** Material Measurement Laboratory, National Institute of Standards and Technology, Gaithersburg, Maryland

**Keywords:** 3D-printed sample mount, forensic glass analysis, primary beam filters, signal-to-noise improvement

## Abstract

Micro X-ray fluorescence spectrometry (μXRF) is a standard technique used for the elemental analysis of glass fragments in forensic casework. The glass specimens encountered in casework are usually small (<1 mm), thin fragments that are partially transparent to the exciting X-ray beam. In addition to providing fluorescence from the small glass fragments, the primary beam X-rays can scatter within the chamber and provide noise in the measurements. To reduce scatter from the sample stage, the fragments are typically mounted on a thin plastic film and raised on an XRF sample cup (≤3 cm in height). However, at these heights, there may still be significant scatter from the sample stage, which adversely affects the signal-to-noise ratio (SNR) and the limit of detection (LOD). A plastic mount was designed and 3D-printed in-house to allow fragments to be raised as high as possible from the sample stage, thereby minimizing stage scatter. Most elements detected in glass showed an improvement in the SNR when using the 3D-printed mount for analyses. The greatest improvement (>30%) was observed for lower atomic number elements (Na and Mg) and higher atomic number elements (Sr and Zr). Another simple method to improve SNR is the use of primary beam filters; when using primary beam filters during analyses, elements with characteristic lines in the high-energy range (Rb, Sr, and Zr) showed the greatest improvement (>70%) in SNR. The impact of both strategies for the improvement of SNR is presented here.

## 1 | INTRODUCTION

Glass is a valuable type of forensic trace evidence that is frequently encountered in crime scenes.^[1,2]^ When glass is broken during the commission of a crime, glass fragments can transfer to an object or person. The majority of glass fragments that are transferred range in size from 1 to 5 mm. However, fragments smaller than 1 mm are more persistent and more common in forensic casework.^[3]^ Forensic analysis of glass involves comparing glass fragments from a known source to fragments from an unknown (questioned) source to determine whether the known and the questioned fragments can be discriminated. Micro X-ray fluorescence spectrometry (μXRF) is a standard technique used for the elemental analysis of glass fragments in casework. Several studies have demonstrated the high discrimination capability of μXRF when comparing glass samples from different sources.^[2,4–6]^

The ASTM standard E2926 provides a test method for the forensic comparison of glass analyzed using μXRF.^[7]^ ASTM E2926 recommends collecting a minimum of three replicate measurements on each of at least three known fragments and a minimum of three replicate measurements on each recovered questioned fragment. Once the known and questioned samples are measured, their spectra are visually inspected for reproducible differences in detected elements. An element is considered detectable (i.e. above the limit of detection, LOD) if its peak has a signal-to-background ratio (SNR) of at least 3; an SNR ≥3 indicates that the element is present at a mass fraction that is greater than the background signal by a statistically significant amount.^[8]^ To express the LOD in mass fraction, a sample with known elemental mass fractions (e.g. a Standard Reference Material, SRM) is required. Equation (1) relates the SNR to the LOD; *c* is the mass fraction for the element of interest.

(1) $$\text{LOD}=\frac{3\times c}{\text{SNR}}.$$

If no differences are found by the visual inspection of the known and questioned spectra, a semi-quantitative approach using element intensity ratios is employed. Although an element may be detectable, there is greater uncertainty in a measurement when the element mass fraction is near the LOD. Therefore, to improve precision and accuracy, only elements that have an SNR of at least 10 (i.e. above the limit of quantitation, LOQ) are typically used for semi-quantitative comparisons with element ratios.^[7,8]^ Equation (2) relates the SNR to the LOQ (in units of mass fraction).

(2) $$\text{LOQ}=\frac{10\times c}{\text{SNR}}.$$

When analyzing thin glass samples (as is often the case with forensic specimens), scatter from the instrument stage can result in a high background, which reduces the SNR and, consequently, yields poorer LODs and LOQs. To reduce the background due to stage scatter, ASTM E2926 suggests raising samples off the stage by using a sample holder and/or a thin supportive X-ray film. However, the standard does not specify how high specimens should be raised from the stage. Typical XRF sample cups used for the analysis of thin samples are 2–3 cm in height; at these heights, there is still significant, detectable scatter from the stage. To minimize stage scatter, a tall (>8 cm), plastic mount was designed and 3D-printed at the National Institute of Standards and Technology (NIST), which allowed samples to be raised higher than traditional XRF sample mounts. Considering the affordability of 3D printers, a 3D-printed sample mount is a simple and practical option to minimize stage scatter and improve the SNR in XRF analyses.

Another method to improve SNRs is through the use of primary beam filters. Filters absorb part of the polychromatic excitation of the X-ray tube and change the energy distribution that excites the sample. Filters can be used to remove interfering lines from the X-ray tube target element and to improve the SNR in a particular region of the spectrum.^[9]^ Yet despite these advantages, filters are not currently utilized by the forensic community.

The aim of this study is to illustrate the magnitude of stage scatter and its effect on SNR. A simple solution involving the development of a 3D-printed sample mount is proposed. Also, the utility of primary beam filters for the improvement of SNR is discussed. Although this study focused on the analysis of small glass fragments, the proposed methods for improving SNR can be applied to any sample that is partially transparent to the primary beam.

## 2 | METHODS

In this study, two μXRF instruments were used, which will be referred to as Instrument 1 and Instrument 2. One of the instruments is equipped with a Rh X-ray tube with an incident angle of ≈50° and a beryllium window with a thickness of 100 μm, poly-capillary optics with a 20 μm spot size, and two silicon drift detectors (SDD), each with an area of 60 mm^2^ and a beryllium window with a thickness of 13 μm. The other instrument is equipped with a Rh X-ray tube with an incident angle of 90° and a beryllium window with a thickness of 125 μm, poly-capillary optics with a 30 μm spot size, one SDD with an area of 50 mm^2^, and a beryllium window with a thickness of 8 μm.

**FIGURE 1:**
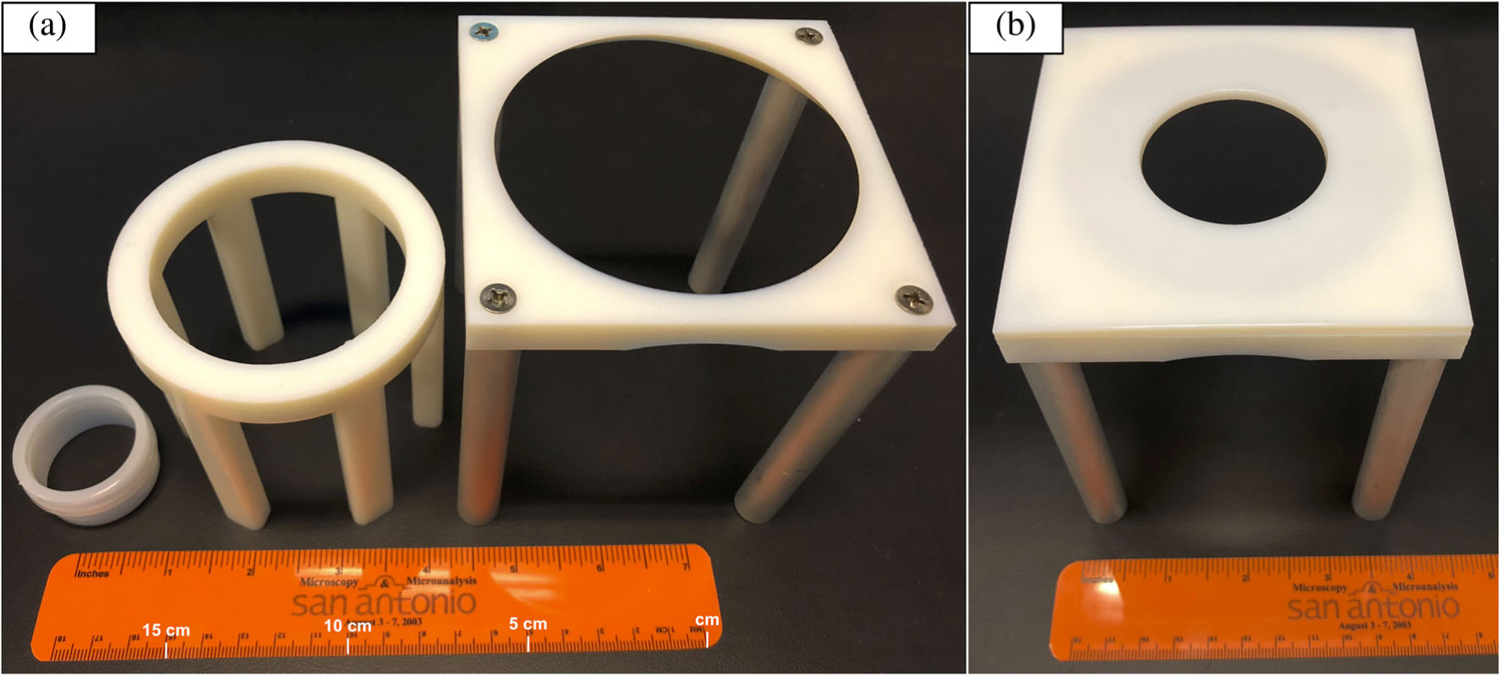
(a) Photo of different mounts used for analysis, from left to right: X-ray fluorescence spectrometry sample cup (2.13 cm in height), Mount 1 (8.2 cm in height), and Mount 2 (11 cm in height). (b) Mount 2 with attachable insert to accommodate smaller films

Small fragments are typically analyzed using XRF sample cups that are ≤3 cm in height. To raise samples higher than traditional XRF sample cups, a plastic sample mount (subsequently referred to as “Mount 1”) with a height of 8.2 cm was 3D-printed at NIST. A second, taller mount (11 cm) was also 3D-printed (“Mount 2”). Figure 1 shows a photo of an XRF sample cup (Chemplex Industries^1^), Mount 1, and Mount 2. For Mount 2, a plastic insert was 3D-printed to accommodate smaller mounting films (Figure 1b).

**FIGURE 2:**
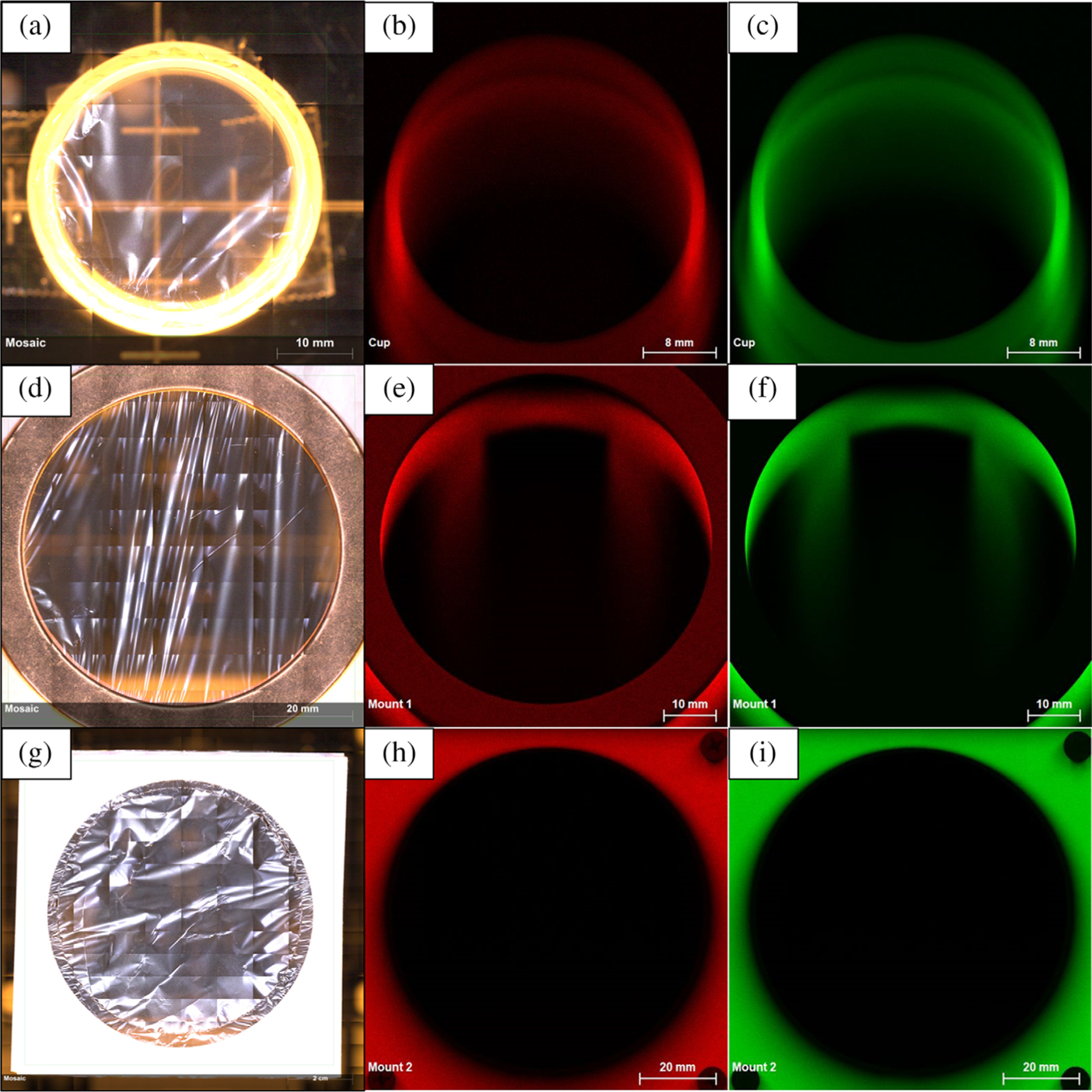
(a–c) X-ray fluorescence spectrometry sample cup with thin film, Rayleigh (Rh, Kα) intensity map (red), and Compton intensity map (green), respectively. (d–f) Mount 1 with thin film, Rayleigh (Rh, Kα) intensity map (red), and Compton intensity map (green), respectively. (g–i) Mount 2 with thin film, Rayleigh (Rh, Kα) intensity map (red), and Compton intensity map (green), respectively. Instrument 1 analysis parameters: 50 kV, 300 μA, both silicon drift detectors, 275 kcps (×1,000 counts per second) pulse throughput, 630 μm Al filter

Since the X-ray beam for Instrument 1 has a 50° incidence, the primary beam can strike the inside wall of the XRF sample cup or the plastic legs of Mount 1, which can contribute to the background scatter. This is not the case for Instrument 2, because the X-ray beam is perpendicular to the sample stage. To explore the potential scatter from each mount, intensity maps were collected across the top surface of each mount using Instrument 1. Figure 2a, d, and g shows a top view of the sample cup, Mount 1, and Mount 2, respectively. A thin film window carrier frame (Chemplex Industries^1^, Palm City, FL, USA) was placed on each mount; a copper ring was placed on top of the thin film frame for Mount (Figure 2d) to hold the frame in place during measurements. Intensity maps for the elastic (Rayleigh) and inelastic (Compton) scatter are shown for the XRF cup (Figure 2b,c), Mount 1 (Figure 2e,f), and Mount 2 (Figure 2h,i). Scatter from the inner wall of the XRF sample cup and the legs of Mount 1 is clearly evident. The square design of Mount 2 allowed the legs to be positioned as far apart as possible, which corrected the scatter issues observed for Mount 1; the metal legs of Mount 2 reduced the printing time.

The NIST Standard Reference Material (SRM®) 1831 was used to determine LOD with SNR and LOD calculated using the procedure outlined in Ernst, et al.^[8]^ A small (<1 mm) SRM 1831 fragment was glued to a thin film frame using Elmer’s washable glue. The glue was smeared on the thin film using a microscope glass cover slip. The LOD was calculated using the certified mass fraction for each element in SRM 1831. For elements that did not have a certified mass fraction, reported values were used.^[10]^ Table 1 lists the mass fractions used to calculate the LOD for each detectable element.

## 3 | RESULTS AND DISCUSSION

Several factors can significantly affect sensitivity, including the X-ray tube window, the type of detector, the detector solid angle and window, and the focusing optics (monocapillary, poly-capillary). However, these parameters are not adjustable by the user. Parameters that can be adjusted include the following: primary beam voltage, current, pulse throughput, and primary beam filters (if available). Lower voltage increases the sensitivity for the low atomic number elements, whereas higher voltage increases the sensitivity of high atomic number elements. Higher current and pulse throughput increase the sensitivity (but also increase the dead time). Primary beam filters increase the sensitivity in a particular region of the spectrum (but decrease the sensitivity in lower energy regions).^[9]^

### 3.1 | Improving SNR by raising samples

**FIGURE 3:**
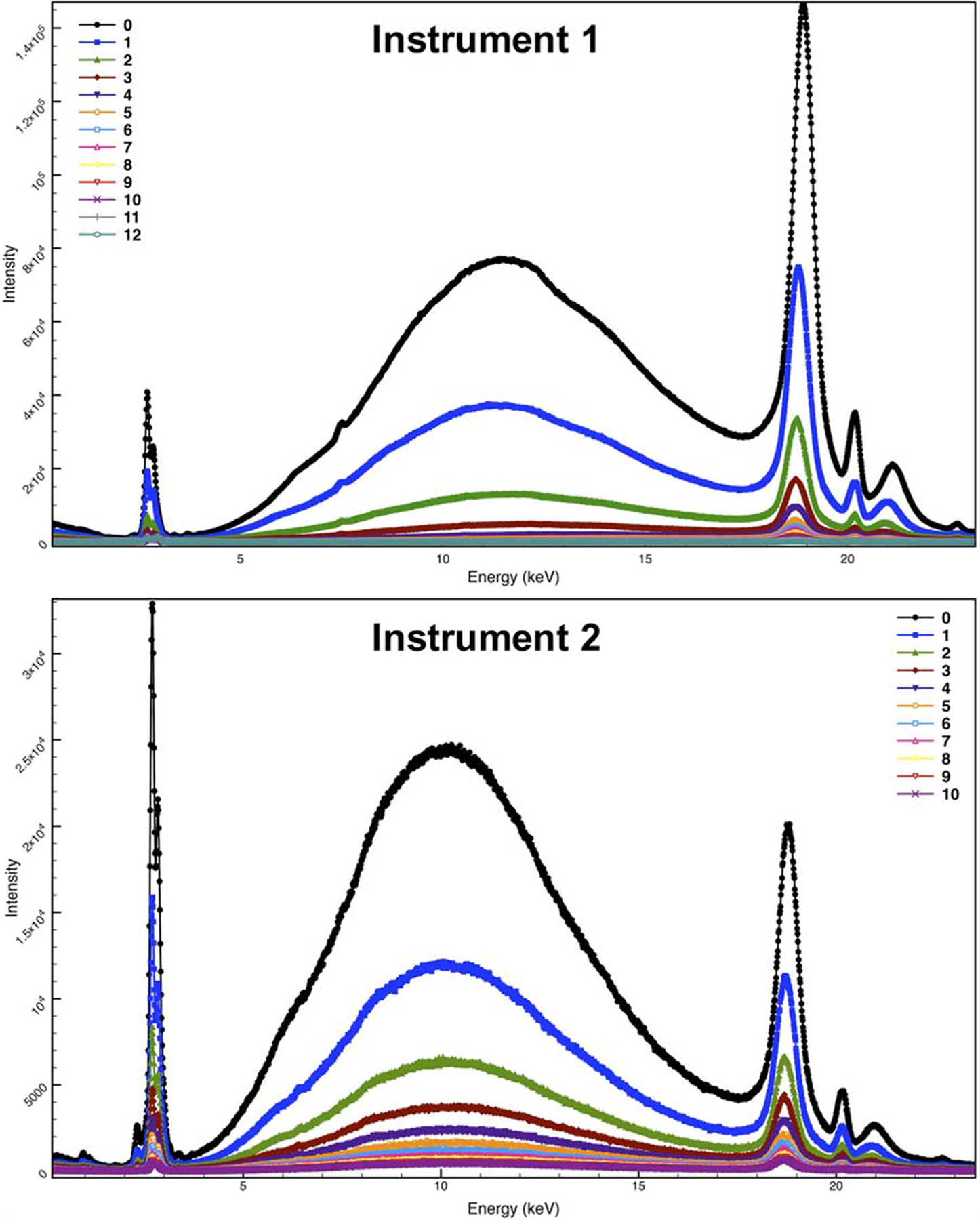
Spectra of sample stage for each instrument; spectrum 0 (black) was collected with the stage in focus and remaining spectra were collected at 1 cm increments below focal point. Instrument 1 analysis parameters: 50 kV, 300 μA, 500 live s, both silicon drift detectors, 130 kcps pulse throughput. Instrument 2 analysis parameters: 50 kV, 100 μA, 1,500 live s, 3.2 μs pulse process time

To illustrate the magnitude of scatter from the plastic stage, each instrument’s plastic stage was analyzed at various heights, beginning at the height in which the stage was in focus and then lowering the stage in 1-cm increments. Figure 3 shows the spectra collected for Instrument 1 (top) and Instrument 2 (bottom). For each plot, the spectrum labeled 0 (shown in black) is the spectrum collected when the stage was in focus. Each subsequently numbered spectrum represents the spectrum collected after lowering the stage below the focal point. For example, spectrum 4 was collected with the stage lowered 4 cm below the focal point; spectrum 8 was collected 8 cm below the focal point and so forth. It is evident that at the heights of typical XRF sample cups (2–3 cm) there is still significant scatter from the stage (spectrum 2, green and spectrum 3, red, for each instrument).

**FIGURE 4:**
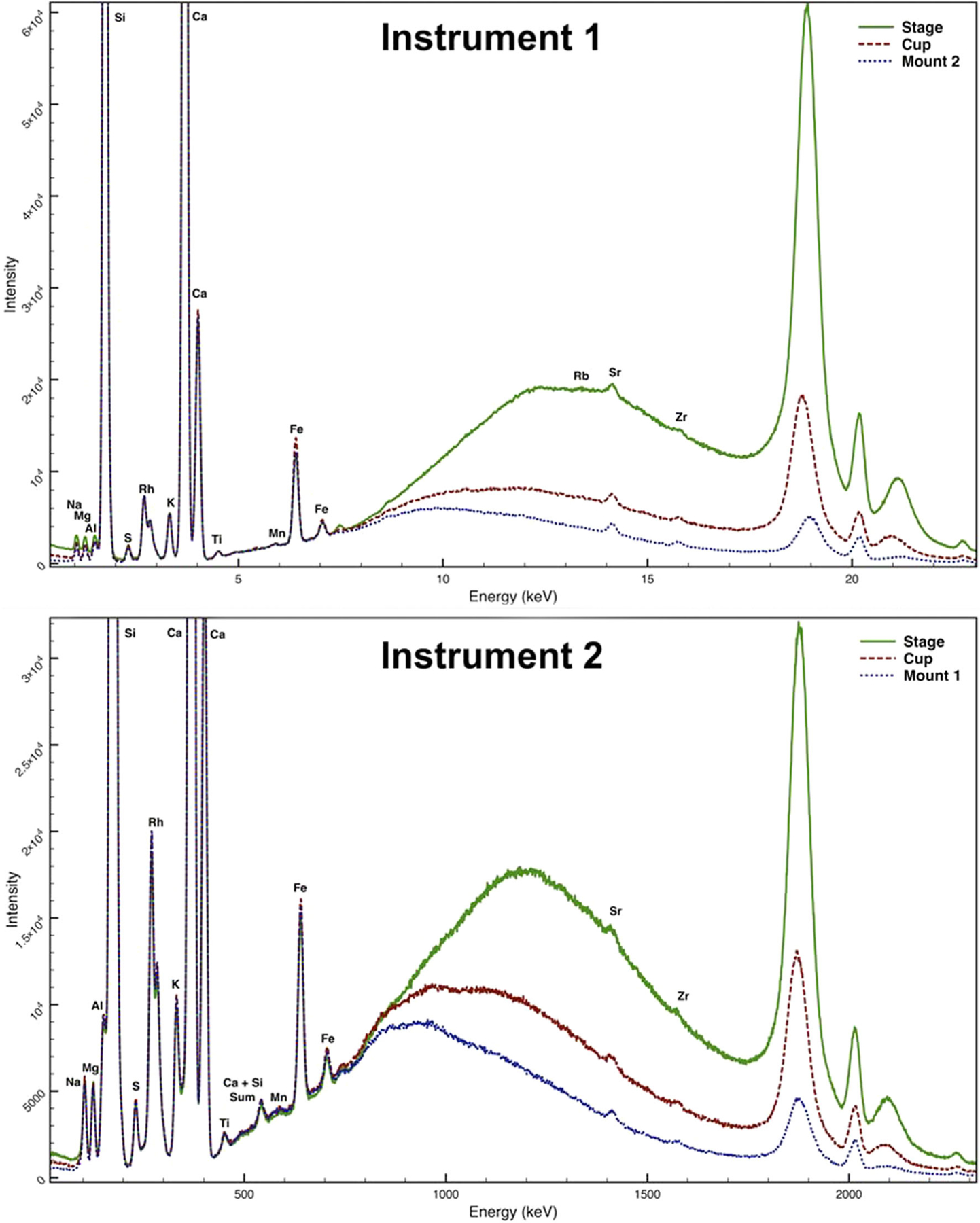
Spectra of small (<1 mm) SRM 1831 fragment analyzed at various stage heights for each instrument: directly on sample stage (green solid line), raised on X-ray fluorescence spectrometry sample cup (red dashed line), and raised on Mount 1 for Instrument 2 and Mount 2 for Instrument 1 (blue dotted line). For clarity, only one replicate measurement is displayed. Analysis parameters are listed in Table 1

To compare the LODs at various stage heights, a small (<1 mm) SRM 1831 fragment was analyzed by placing the thin film frame with the glued fragment directly on the sample stage, raised on an XRF sample cup (2.13 cm in height), and raised on a 3D-printed mount (Mount 1 for Instrument 2 and Mount 2 for the Instrument 1). Figure 4 shows the spectra collected at each stage height for both instruments. A reduction in background scatter is seen in the low-energy region (<1.5 keV) and mid- to high-energy region (>8 keV) of the spectrum. The LODs (average of four replicate measurements) in micrograms per gram are reported in Table 1 for both instruments at each of the three heights. Elements in the lower-energy (<2.5 keV) and higher-energy (>13.5 keV) regions showed an improvement in LOD when the glass fragment was analyzed on the mount as opposed to an XRF sample cup, whereas elements with lines in the mid-energy region showed little to no difference in LOD. The greatest improvement (>30%) was observed for low and high atomic number elements (Na, Mg, Sr, and Zr) for Instrument 1 and high atomic number elements (Sr and Zr) for Instrument 2. The improvement for low atomic number elements is especially advantageous since these elements exhibit poor sensitivity because of their low excitation probability, low fluorescence yield, and strongly absorbed radiation.^[11]^ On the other hand, higher energy X-rays have larger penetration depths, which can adversely affect the LOD for high atomic number elements when analyzing a fragment that is not infinitely thick for a particular characteristic X-ray. Therefore, improvement of LOD for high atomic number elements is particularly beneficial when analyzing small glass fragments.^[8]^

Potassium, calcium, titanium, manganese, and iron all showed little to no difference in LOD. In some cases, a small increase in LOD (<10%) was observed when the glass fragment was analyzed raised on the mount as opposed to on the XRF sample cup. However, it should be noted that in these cases, the LOQ was still well below the expected mass fraction of K, Ca, Ti, and Fe in soda-lime float glass, the most common type of glass encountered in casework. Thus, the slightly poorer LODs are not expected to adversely affect analyses of soda-lime float glass in forensic casework.^[12]^ On the other hand, Mn can be present at mass fractions less than 10 μg·g^−1^ in soda-lime glass.^[12]^ Rubidium showed a moderate improvement (<20%) when analyzed on the stage rather than raised on a mount; however, in all cases, Rb was close to or below the LOQ. Similarly to Mn, Rb has been reported to be present at low mass fractions in soda-lime float glass (as low as 0.3 μg·g^−1^).^[12]^ To improve the SNR for elements such as Mn and Rb, the acquisition time can be increased and/or primary beam filters can be used; the latter is discussed in the following section. If, however, the mass fraction for an element remains below the LOQ, it should be excluded from pairwise comparisons using element intensity ratios, as discussed in ASTM E2926.^[7]^

Overall, simply raising specimens farther from the stage improves the SNR for most elements that are typically detected in soda-lime float glass using μXRF. Moreover, the greatest improvements were seen for elements that often have poorer LODs (elements with lines in the low-energy and high-energy regions). Since inexpensive 3D printers are readily available, a printed mount provides a simple and practical solution for reducing stage scatter.

### 3.2 | Improving SNR by using primary beam filters

**FIGURE 5:**
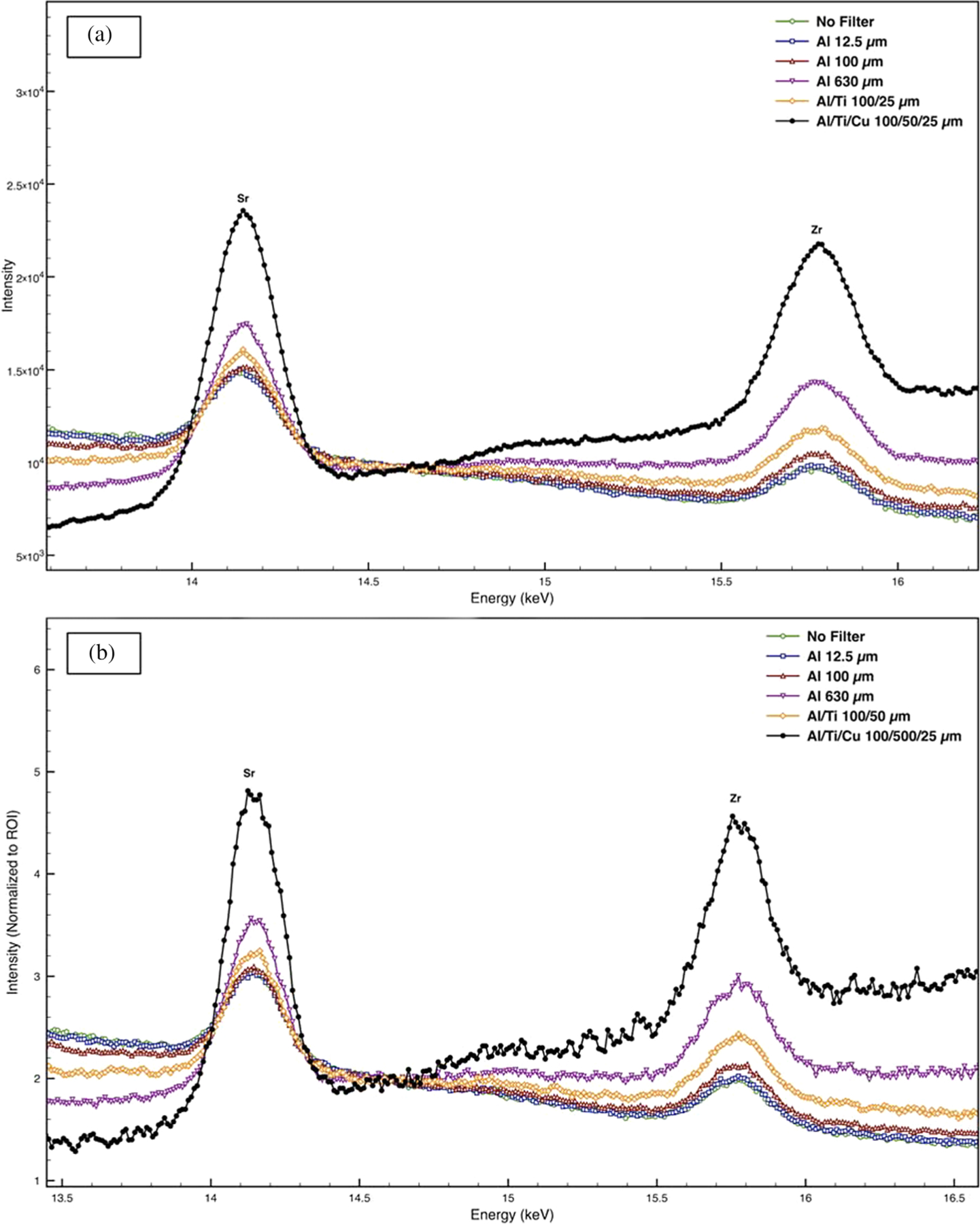
Bulk SRM 1831 analyzed with and without a primary beam filter. Spectra were collected using (a) equal counts in a region of interest (500,000 counts in the region 14.34 keV–14.84 keV) and (b) equal acquisition times (1,000 live seconds; note spectra were normalized to the integrated area in the region of interest 14.34 keV–14.84 keV). For clarity, only one replicate measurement is displayed. Analysis parameters: 50 kV, 200 μA, two silicon drift detectors, 130 kcps pulse throughput

Another simple, yet underutilized, method to improve the SNR is the use of primary beam filters. Instrument 1 is equipped with five filters of different composition and/or thickness: Al (12.5 μm thickness), Al (100 μm thickness), Al (630 μm thickness), Al and Ti (100 and 25 μm thickness, respectively), and Al, Ti, and Cu (100, 50, and 25 μm thickness, respectively). To illustrate the improvement in SNR, a bulk SRM 1831 fragment was analyzed using either no filter or one of the five available filters. Since the count rate decreases with increasing filter thickness, the spectra were collected with a different acquisition time was used for each filter (Table 2) so equal counts (500,000 counts) were collected in a region of interest (14.34–14.84 keV). The region of interest (ROI) was selected to illustrate the improvement for the Sr Kα (14.16 keV) and the Zr Kα (15.78 keV) lines. Table 2 lists the LODs for all detectable elements. The best LOD was observed using no filter for elements with lines in the low-energy region (Na, Mg, Al, and K) and the thickest filter for elements with lines in the high-energy region (Rb, Sr, and Zr); elements with lines in the mid-energy region showed the best LOD with one of the four remaining filters. Rubidium, strontium, and zirconium showed the greatest improvement in LOD (>70%) when using a filter during analysis. Figure 5a clearly illustrates the improvement in SNR for Sr and Zr when using a primary beam filter compared with no filter. The large improvement in Rb LOD is especially significant since, as mentioned in the previous section, Rb can be present at mass fractions as low as 0.3 μg·g^−1^ in soda-lime float glass.^[12]^ Titanium, manganese, and iron showed a significant improvement in LOD as well (20, 27, and 58%, respectively) when using a filter versus no filter. Like Rb, and Mn can be present at fairly low mass fractions (<10 μg·g^−1^) in soda-lime glass. While for Ti and Fe, the LOQs were well below the expected mass fractions of these elements in soda-lime float glass, regardless of which filter was used.^[12]^

As discussed earlier, the use of thicker filters results in lower count rates; therefore, longer acquisition times were used with the thicker filters to ensure equal counts were collected in the selected ROI. If constant acquisition time is used for all filters, there is a deterioration in precision with increasing filter thickness. Figure 5b shows the spectra collected using different filters with constant time (1,000 s live time). For comparison, the spectra were normalized to the ROI 14.34–14.84 keV. Figure 5b clearly shows an increase in noise when using the thickest filter (Al/Ti/Cu 100/50/25 μm) as a result of the lower count rate. The decrease in count rate ultimately led to a poorer SNR and LOD for Rb and Zr (Table 3) when using the thickest filter. This is in contrast to the results presented in Table 2, which show a > 70% improvement in LOD for Rb and Zr when using the thickest filter. Moreover, when using constant time to compare the performance of different filters, the improvement in LODs was less pronounced than the results presented in Table 2, with only Fe and Rb showing an improvement >20%.

The results in Tables 2 and 3 demonstrate that it is necessary to acquire for longer times to avoid the deterioration in precision due to lower count rates when using primary beam filters. As such, there is a trade-off between shorter acquisition times and using a filter to reduce the background signal. However, the lower count rates when using filters permit the use of a higher beam current while still maintaining a reasonable dead time. As shown in Table 2, a higher beam current was used for thicker filters, which reduced the acquisition time required to obtain 500,000 counts in the selected ROI. The beam current for each filter was selected to obtain a dead time of approximately 4%. While keeping the dead time constant (but using a different beam current), the acquisition time using the thickest filter was 4.4 times greater than that of no filter (3,453 s live time vs. 786 s live time, respectively), which resulted in a 3.2-fold decrease (improvement) in LOD for Zr. To obtain a similar LOD improvement using no filter, an acquisition time of approximately 8,050 s live time (3.2^2^ × 786 ≈ 8,050, refer to Ernst et al.^[8]^ for calculations) would be necessary, which is more than double the acquisition time used for the thickest filter. Thus, the higher beam currents that can be used with primary beam filters result in a more efficient improvement in LOD. Nonetheless, to minimize the overall analysis time, filters are best suited for improving the SNR of target elements that are below the LOD or LOQ. For example, if an analyst is unsure of the presence of an element because its peak has an SNR < 3, the glass fragment(s) may be reanalyzed with a filter to improve the SNR, confirm the presence of the element, and include the element in spectral overlay comparisons. Similarly, if the element is detectable (>LOD), but less than the LOQ, a filter can improve the SNR so the element may be included in semi-quantitative comparisons (using intensity ratios).

## 4 | CONCLUSIONS

This study focused on the improvement of SNR for the forensic analysis of glass using μXRF. Two simple methods to improve SNRs were proposed: the first method involved the development of a tall, 3D printed mount to raise small samples as high as possible, thereby minimizing stage scatter. The second method involved the use of primary beam filters, which absorb part of the polychromatic primary X-rays from the X-ray tube, altering the energy distribution that excites the sample. Ultimately, the SNR was varied by adjusting the X-ray beam current, the acquisition time, and the shape of the background (by raising the samples and/or using filters).

It was demonstrated that stage scatter is significant for thin samples, even when samples are mounted on typical XRF sample cups. Scatter can be observed from the inner wall of the XRF sample cup for Instrument 1, which has an X-ray beam that is not perpendicular to the sample stage. Simply raising samples farther from the stage using a 3D-printed mount resulted in an improvement of the SNR for most elements in SRM 1831. Moreover, the greatest improvement was observed for elements that can often suffer from poor LODs: elements with lines in the low-energy region (Na and Mg), which have a low excitation probability, low fluorescence yield, and strongly absorbed radiation; and elements with lines in the high-energy region (Sr and Zr), which have larger penetration depths and, consequently, have low fluorescence intensities in samples that are not infinitely thick. Potassium, calcium, titanium, manganese, and iron showed little to no differences in LOD when analyzed on a mount versus directly on the sample stage, whereas Rb showed a moderate improvement in LOD when analyzed directly on the stage. Since Mn and Rb have reported mass fractions that are below 10 μg·g^−1^ in soda-lime glass, maximizing the SNR (e.g. through the use of filters) for these two elements is advantageous for the forensic analysis of soda-lime glass. Note that the detection limits, the relative improvement of SNR results, and the shape of the scatter spectrum will vary with the composition, morphology, and mass-thickness of the sample as seen by the primary beam and X-ray detectors. Additionally, the transmission effect of the optical component (e.g. poly-capillary lens) will affect the shape of the scatter spectrum.^[13]^ Thus, the specific LODs and LOQs listed here only apply to these instruments and to this glass composition (SRM 1831) and particle, but they serve as demonstrations of improving SNRs.

The use of primary beam filters can improve LODs for X-ray lines with energies >4 keV; elements with X-ray lines <4 keV showed little to no improvement. Since using filters results in lower count rates, longer acquisition times are necessary to obtain good counting statistics and avoid deterioration in precision. When using primary beam filters, Rb, Sr, and Zr showed the largest improvements in LOD (>70%). Titanium, manganese, and iron also showed significant improvement in LOD (>20%). However, Ti and Fe are usually present in soda-lime glass at mass fractions that are well above the LOQ; though, other types of glass may have lower mass fractions of these two elements. As noted above, the improvements in the Mn and Rb LODs are especially significant, since both elements can be present at very low mass fractions (<1 μg·g^−1^). In addition, the LOD improvement for elements with lines in the high-energy region (Rb, Sr, and Zr) is valuable, since these elements can have low fluorescence intensities in thin samples (due to the larger penetration depth of higher energy lines). For elements with low mass fractions (Mn and Rb) and/or high fluorescence energies (Rb, Sr, and Zr), using a filter with longer acquisition times can increase the SNR so that the element mass fraction is above the LOQ and can be used in semi-quantitative comparisons. It should be noted that an analyst can select or design primary beam filters to enhance the sensitivity of specific elements (e.g. the elements that are most discriminating), thereby optimizing μXRF for forensic glass analysis. It is worth noting that lowering the X-ray beam voltage can improve the sensitivity of elements with lines in the low-energy region. Thus, an analyst can use different voltages to improve the SNR in different regions of the spectrum, without the need for primary beam filters in the low-energy or mid-energy regions.

Based on the results of this study, it is suggested that small samples be analyzed first with no filter and raised on a tall mount, such as the one developed here. The known and questioned sample should be compared by visual inspection of their spectra using only elements above the LOD (SNR > 3). The samples can be reanalyzed with a filter, with longer acquisition times, and/or with multiple detectors, if available, to improve the SNR of elements that appear to be present but are below the LOD. If there are no differences in detected elements between the known and questioned sample, a semi-quantitative approach can be employed using intensity ratios with elements above the LOQ (SNR > 10). Reanalyzing the samples with a filter, longer acquisition times, and/or multiple detectors can improve the SNR of elements below the LOQ. Improved SNRs result in superior LODs and LOQs, which can increase the number of elements that can be used for pairwise comparisons and, ultimately, improve the discrimination potential of μXRF for the analysis of glass.

Note that raising a specimen far above the stage can improve the analysis of any type of sample (not only glass) that is partially transparent to the primary beam or small relative to the primary beam, since this will ensure that the background scatter is predominantly from the specimen itself and not the instrument. As particles approach bulk thickness (opaque to the primary beam), the stage plays an insignificant role and raising the specimen above the stage is not necessary, but filters may still be useful for improving the SNR.

## Figures and Tables

**TABLE 1:** Average limit of detection (LOD) (μg·g^−1^, *n* = 4 replicates) ± one standard deviation for SRM 1831 analyzed at different heights

Element	Mass fraction (μg·g^−1^)	Instrument 1			Instrument 2		
		Stage	Cup	Mount 2	Stage	Cup	Mount 1
Na	98,816^a^	1,859 ± 34	1,152 ± 24	911 ± 27	1,249 ± 21	1,096 ± 20	1,015 ± 7.2
Mg	21,200^a^	381 ± 2.7	248 ± 4.1	214 ± 7.2	298 ± 5.4	265 ± 1.8	250 ± 3.3
Al	6,380^a^	151 ± 1.9	124 ± 1.3	121 ± 4.2	187 ± 8.1	191 ± 3.6	185 ± 1.6
K	2,740^a^	12 ± 0.06	11 ± 0.09	12 ± 0.20	30 ± 0.41	29 ± 0.12	29 ± 0.11
Ca	58,600^a^	6.3 ± 0.02	5.8 ± 0.01	6.0 ± 0.12	13 ± 0.06	13 ± 0.05	12 ± 0.04
Ti	114^a^	2.9 ± 0.05	2.7 ± 0.05	2.8 ± 0.07	5.5 ± 0.20	5.2 ± 0.21	5.3 ± 0.21
Mn	15^b^	1.2 ± 0.02	1.3 ± 0.11	1.3 ± 0.06	3.5 ± 0.39	3.8 ± 0.56	3.8 ± 0.49
Fe	608^a^	1.8 ± 0.01	1.8 ± 0.004	1.8 ± 0.02	4.4 ± 0.02	4.3 ± 0.01	4.4 ± 0.02
Rb	6.11^b^	1.6 ± 0.28	[2.1 ± 0.32]	[1.9 ± 0.38]	N/A	N/A	N/A
Sr	89.12^b^	3.7 ± 0.09	2.3 ± 0.05	2.0 ± 0.07	14 ± 1.0	11 ± 1.4	8.9 ± 0.75
Zr	43.36^b^	4.8 ± 0.38	2.4 ± 0.13	1.9 ± 0.12	[25 ± 7.2]	12 ± 0.80	8.6 ± 0.93

*Note:* Bracketed value indicates SNR < 10 (below LOQ); N/A indicates SNR < 3 (below LOD). Instrument 1 analysis parameters: 50 kV, 300 μA, 1,000 s live, two silicon drift detectors, 130 kcps pulse throughput, resolution ≈ 143 eV FWHM Mn K_α_. Instrument 2 analysis parameters: 50 kV, 300 μA, 1,000 s live, 3.2 μs pulse processing time, resolution ≈ 142 eV FWHM Mn K_α_.

a Certified mass fraction (National Institute of Standards and Technology).

b Reported mass fraction.^[10]^

**TABLE 2:** Average limit of detection (LOD) (μg·g^−1^, *n* = 10 replicates) ± one standard deviation for Bulk SRM 1831 analyzed using different filters and constant counts in region of interest

	No filter	Al (12.5 μm)	Al (100 μm)	Al (630 μm)	Al and Ti (100 and 25 μm)	Al, Ti, and Cu (100, 50, and 25 μm)
Live time (ls)	786	640	484	969	523	3,453
Current (μA)	200	250	400	600	600	600
Na	**1,401 ± 14**	7,287 ± 212	21,867 ± 2,374	[65,340 ± 48,173]	[40,807 ± 13,242]	[43,280 ± 8,922]
Mg	**311 ± 4.1**	1,517 ± 32	4,925 ± 726	[15,180 ± 6,993]	[10,536 ± 3,334]	[11,666 ± 4,408]
Al	**163 ± 3.1**	542 ± 25	1,595 ± 255	[4,522 ± 2,857]	[3,288 ± 1,080]	[6,277 ± 4,264]
K	**14 ± 0.11**	15 ± 0.09	26 ± 0.26	57 ± 1.2	41 ± 0.77	65 ± 1.2
Ca	7.4 ± 0.03	**7.1 ± 0.04**	11 ± 0.12	26 ± 0.29	18 ± 0.18	30 ± 0.20
Ti	3.4 ± 0.04	3.0 ± 0.05	**2.7 ± 0.07**	7.1 ± 0.41	4.7 ± 0.16	8.8 ± 0.40
Mn	1.7 ± 0.11	1.7 ± 0.14	1.3 ± 0.04	1.5 ± 0.13	**1.2 ± 0.09**	1.4 ± 0.09
Fe	2.3 ± 0.01	2.1 ± 0.01	1.3 ± 0.01	1.2 ± 0.01	**0.9 ± 0.01**	1.2 ± 0.01
Rb	1.8 ± 0.27	[1.9 ± 0.28]	1.6 ± 0.14	0.9 ± 0.09	1.2 ± 0.17	**0.4 ± 0.02**
Sr	1.9 ± 0.04	1.8 ± 0.03	1.6 ± 0.02	0.9 ± 0.01	1.3 ± 0.01	**0.5 ± 0.003**
Zr	1.6 ± 0.03	1.6 ± 0.03	1.4 ± 0.03	0.8 ± 0.01	1.1 ± 0.03	**0.5 ± 0.004**

*Note:* Best (lowest) LOD is indicated with bold font for each element. Bracketed value indicates SNR < 10 (below LOQ). Analysis parameters: 50 kV, 200–600 μA, two silicon drift detectors, 130 kcps pulse throughput; note that the acquisition time (seconds live) varied for each filter.

**TABLE 3:** Average limit of detection (LOD) (μg·g^−1^, *n* = 5 replicates) ± one standard deviation for Bulk SRM 1831 analyzed using different filters and constant acquisition time

	No filter	Al (12.5 μm)	Al (100 μm)	Al (630 μm)	Al and Ti (100 and 25 μm)	Al, Ti, and Cu (100, 50, and 25 μm)
Na	**1,235 ± 7.9**	6,964 ± 324	20,800 ± 2,820	[183,242 ± 201,565]	[55 428 ± 21,387]	[71,764 ± 12,692]
Mg	**278 ± 2.2**	1,401 ± 47	4,851 ± 603	[24,666 ± 18,842]	[9,693 ± 2,343]	[28,390 ± 27,193]
Al	**145 ± 2.1**	481 ± 30	1,588 ± 247	[5,153 ± 906]	[2,693 ± 411]	N/A
K	**13 ± 0.05**	14 ± 0.13	25 ± 0.18	96 ± 2.4	49 ± 1.2	206 ± 9.3
Ca	6.6 ± 0.03	**6.4 ± 0.04**	11 ± 0.08	43 ± 0.46	22 ± 0.30	97 ± 3.0
Ti	3.0 ± 0.05	2.7 ± 0.04	**2.6 ± 0.06**	13 ± 0.81	5.8 ± 0.28	28 ± 5.6
Mn	1.5 ± 0.08	1.7 ± 0.10	**1.3 ± 0.07**	2.6 ± 0.43	1.4 ± 0.15	4.7 ± 0.73
Fe	2.0 ± 0.01	1.9 ± 0.01	1.3 ± 0.004	1.9 ± 0.02	**1.1 ± 0.01**	3.7 ± 0.11
Rb	1.7 ± 0.23	1.6 ± 0.15	1.6 ± 0.25	**1.3 ± 0.15**	1.3 ± 0.43	1.4 ± 0.57
Sr	1.7 ± 0.02	1.7 ± 0.02	1.6 ± 0.02	1.5 ± 0.01	1.6 ± 0.02	**1.4 ± 0.01**
Zr	1.4 ± 0.02	1.4 ± 0.03	1.4 ± 0.02	**1.4 ± 0.03**	1.4 ± 0.03	1.5 ± 0.02

*Note:* Best (lowest) LOD is indicated with bold font for each element. Bracketed value indicates SNR < 10 (below LOQ); N/A indicates SNR < 3 (below LOD). Analysis parameters: 50 kV, 200 μA, 1,000 s live time, two silicon drift detectors, 130 kcps pulse throughput.

## ENDNOTE

1 Certain commercial equipment, instruments, or materials are identified in this paper in order to specify the experimental procedure adequately. Such identification is not intended to imply recommendation or endorsement by the National Institute of Standards and Technology, nor is it intended to imply that the materials or equipment identified are necessarily the best available for the purpose.

## References

[1] Peterson J, National Institute of Justice, U. S. G. P. Office, Washington D. C., 1974.

[2] Buscaglia J, Anal. Chim. Acta 1994, 288, 17.

[3] Rupert K, Ho M, Trejos T, JASTEE 2018, 8, 16–33.

[4] Trejos T, Koons R, Becker S, Berman T, Buscaglia J, Duecking M, Eckert-Lumsdon T, Ernst T, Hanlon C, Heydon A, Mooney K, Nelson R, Olsson K, Palenik C, Pollock EC, Rudell D, Ryland S, Tarifa A, Valadez M, Weis P, Almirall J, Anal. Bioanal. Chem 2013, 405, 5393–5409. 23673570 10.1007/s00216-013-6978-y

[5] Trejos T, Koons R, Weis P, Becker S, Berman T, Dalpe C, Duecking M, Buscaglia J, Eckert-Lumsdon T, Ernst T, Hanlon C, Heydon A, Mooney K, Nelson R, Olsson K, Schenk E, Palenik C, Pollock EC, Rudell D, Ryland S, Tarifa A, Valadez M, van Es A, Zdanowicz V, Almirall J, J. Anal. At. Spectrom 2013, 28, 1270–1280.

[6] Naes BE, Umpierrez S, Ryland S, Barnett C, Almirall JR, Spectrochim. Acta B 2008, 63, 1145–1150.

[7] ASTM E2926–17. ASTM International West Conshohocken, PA. 2017.

[8] Ernst T, Berman T, Buscaglia J, Eckert-Lumsdon T, Hanlon C, Olsson K, Palenik C, Ryland S, Trejos T, Valadez M, Almirall JR, X-Ray Spectrom 2014, 43, 13.

[9] Grieken REV, Markowicz AA, Handbook of X-Ray Spectrometry, Marcel Dekker, Inc., New York, NY 2002.

[10] ASTM E2330–12. ASTM International West Conshohocken, PA. 2012.

[11] Haschke M, Laboratory Micro-X-Ray Fluorescence Spectroscopy: Instrumentation and Applications, Springer, New York, NY 2014.

[12] ASTM E2927–16e1. ASTM International West Conshohocken, PA. 2016.

[13] Gormley J, Jach T, Steel E, Xiao Q-F, X-Ray Spectrom 1999, 28, 115–120.

